# A case of nephrotic syndrome showing contemporary presence of apolipoprotein E2 homozygote glomerulopathy and membranous nephropathy-like findings modified by apolipoprotein E Toyonaka 

**DOI:** 10.5414/CNCS109509

**Published:** 2018-11-30

**Authors:** Hisako Hirashima, Toshiyuki Komiya, Naoya Toriu, Shigeo Hara, Akira Matsunaga, Takao Saito, Eri Muso

**Affiliations:** 1 Division of Nephrology, Kansai Electric Power Hospital,; 2 Kansai Electric Power Medical Research Insitute, Osaka,; 3 Department of Pathology, Kobe University School of Medicine, Hyogo,; 4 Department of Laboratory Medicine, Faculty of Medicine, Fukuoka University,; 5 Sanko Clinic, Fukuoka, Japan,; 6 Division of Nephrology and Dialysis, Kitano Hospital, Tazuke Kofukai Medical Research Insitute, Osaka, and; 7 Department of Pathological Diagnosis, Kyoto University Hospital, Kyoto, Japan

**Keywords:** apolipoprotein E Toyonaka, apolipoprotein E2/2, type III hyperlipoproteinemia, apolipoprotein E deposition, membranous nephropathy, lipoprotein glomerulopathy

## Abstract

A 79-year-old man was admitted to our hospital for proteinuria due to nephrotic syndrome. Renal biopsy revealed focal sclerosis and foam cell infiltration in the glomerulus. In addition, electron microscopic findings (EM) revealed peculiar electron-dense deposits (EDDs) in both sides of the glomerular basement membrane. Although subepithelial deposits had spike formation highly resembling those seen in membranous nephropathy (MN), immunoglobulins and complements were not identified by immunofluorescence study, and microbubbles appeared in high magnification of EM different from the immune disease. The analysis of apolipoprotein (Apo) E showed an elevated concentration of plasma ApoE. The phenotype, genotype, and DNA sequence studies revealed homozygous ApoE2/2 and a novel missense mutation called ApoE Toyonaka (Ser197Cys). This case may confirm the independent responsibility of ApoE2/2 and ApoE Toyonaka for ApoE2 homozygote glomerulopathy and MN-like EDD findings, respectively.

## Introduction 

Hyperlipoproteinemia (HLP) is sometimes associated with glomerular injuries due to apolipoprotein (Apo) E, one of the important components of lipoprotein, also called lipoprotein glomerulopathy (LPG) [1] and ApoE2 homozygote glomerulopathy. The former is developed with various heterozygous ApoE variants like ApoE Sendai [2] and ApoE Kyoto [3] and shows typical histological findings of lipoprotein thrombi in dilated glomerular capillaries [1]. The latter is characterized by type III HLP with homozygous ApoE2/2 and shows sclerotic lesions and foam cells in the glomerulus [4]. In fact, among the three representative isoforms of apolipoproteins, ApoE2 has a low-binding affinity to low-density lipoprotein receptor of only 2% compared to that of wild-type apolipoprotein, ApoE3 [5], and, occasionally, ApoE2/2 develops type III HLP. 

We recently encountered a case of nephrotic syndrome (NS) accompanying type III hyperlipidemia. The analysis of *APOE* gene revealed not only ApoE2/2 homozygosity but also a novel mutation named ApoE Toyonaka [6]. The pathological findings were the association between ApoE2 homozygote glomerulopathy and membranous nephropathy (MN) like capillary wall lesion. The peculiar clinical and pathological characteristics suggested a new form of ApoE-related glomerular disease other than LPG and ApoE2 homozygote glomerulopathy, which can be induced by the combination of a novel ApoE mutation and homozygous E2/2. 

## Case presentation 

### Patient 

A 79-year-old man was admitted to the Department of Nephrology, Kansai Electric Power Hospital on January 14, 2014, for proteinuria. He had been followed up for hypertension and dyslipidemia in a local clinic since the age of 50. He had experienced several vascular events, such as total occlusion in the common iliac artery at the age of 63 and two histories of cerebral infarction at the ages of 70 and 77. He had 30 years of smoking history but no alcohol habit. He had no family history of kidney disease except for one cousin showing end-stage renal disease of unknown etiology. During his initial admission to our hospital, his urine protein level was 5.4 g/g Cr, and serum albumin was 3.2 g/dL; he was discharged because his condition was stable. Ten months later, edema of his lower limbs worsened, and renal biopsy was performed on his second admission. 

On the second hospitalization, physical examination showed the following: height of 169 cm, weight of 70 kg, body mass index of 24.5, and blood pressure of 180/88 mmHg. Laboratory findings revealed a nephrotic range of urine protein, 9.15 g/g Cr accompanying microscopic hematuria of dysmorphic 10 – 19 RBC/HPF, and total serum protein and albumin of 4.8 and 1.8 g/dL, respectively. Serum creatinine was 0.95 mg/dL, AST was 29 IU/L, ALT was 14 IU/L, and ALP was 188 IU/L. Serological examination revealed serum IgG, IgA, and IgM of 875, 344, and 52 mg/dL, respectively. Antinuclear and antineutrophil cytoplasmic antibodies were negative. 

Lipid analysis revealed elevated total cholesterol (259 mg/dL), triglyceride (376 mg/dL), and LDL cholesterol (167 mg/dL) levels, and a low HDL cholesterol level (45 mg/dL). The serum ApoE level was 13.6 mg/dL (normal range: 2.8 – 4.6 mg/dL). An oral glucose tolerance test showed no abnormality, and his hemoglobin A1c was 5.7%. Although hypertriglyceridemia was marked, systemic abnormalities characteristic of hyperlipidemia, such as corneal opacities, xanthoma, and Achilles tendon hypertrophy, were not found. Moreover, the patient had no family history of hyperlipidemia and diabetes mellitus. 

### Renal biopsy 

**Figure 1.**
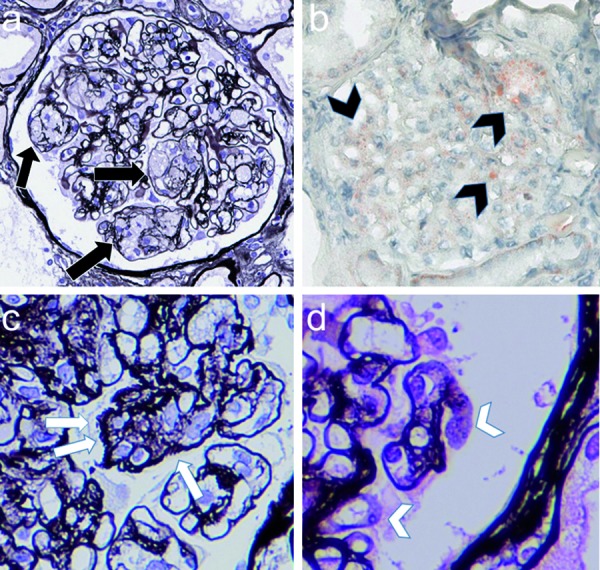
Light microscopic findings. a: Most glomeruli showed segmental infiltration of foam cells in the capillary lumen (black arrows). b: Sudan III-positive staining for lipid (black arrows) is observed in the mesangium and infiltrating foam cells (original magnification × 400). c: In higher magnification, PAM staining revealed partial double contour formation and spike-like lesions (white arrows). d: PAM staining revealed partial bubble-type appearance (white arrows).

**Figure 2.**
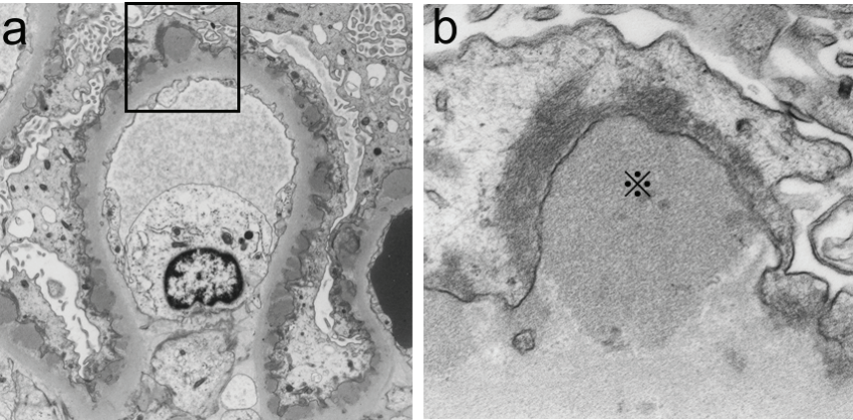
Electron microscopic findings. a: EDDs were observed in subepithelial lesion with membranous nephropathy-like spike formation of the basement membrane (× 2,500). b: Highly-magnified subepithelial position (※) (× 200,000).

**Figure 3.**
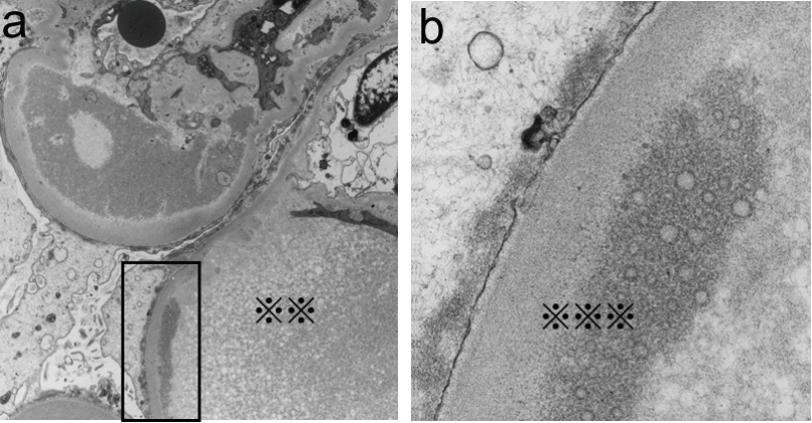
Electron microscopic findings. a: EDDs with various sizes and densities were observed also in the subendothelial lesions. Severe effacement of podocytes was noted (× 2,500). The capillary lumen was filled with lipid deposits that did not show a lamellar formation (※※). b: In higher magnification, EDDs with a high density consist of microbubbles or microcysts (※※※) (× 200,000).

Light microscopy (LM) revealed that the specimens contained 27 glomeruli, 8 of which showed global sclerosis (29.6%). Most glomeruli showed segmental sclerosis, infiltration of foam cells containing lipid deposits in capillary lumen (Figure 1a). In periodic methenamine silver (PAM)-stained sections, spike formation and bubbling were detected on the glomerular basement membrane (GBM) (Figure 1c, d). Mild to severe arteriosclerosis and arteriolosclerosis were also seen. Fibrosis was observed in ~ 20% of the whole interstitial area. Intriguingly, Sudan III staining was strongly positive not only in the mesangial and infiltrating foam cells but also in the focal capillary lumen in the glomerulus (Figure 1b). Immunofluorescence (IF) studies demonstrated subtle granular staining for IgG along the capillary wall; however, C3 was not detected (data not shown). Electron microscopic (EM) findings analysis showed electron-dense deposits (EDDs) with a variety of densities and distributions. Highly-dense deposits accompanied by spike formation identical to MN were seen in the subepithelial area (Figure 2a, b). In the subendothelial area, highly dense EDDs were detected but in higher magnification, and microbubbles were contained (Figure 3a, b). Effacement of foot process was also accompanied with these depositions. In addition, EM findings demonstrated that the capillary lumina was filled with lipid deposits that did not show lamellar formation similar to “lipid thrombi” in LPG. 

### Analysis of ApoE mutation 

**Figure 4.**
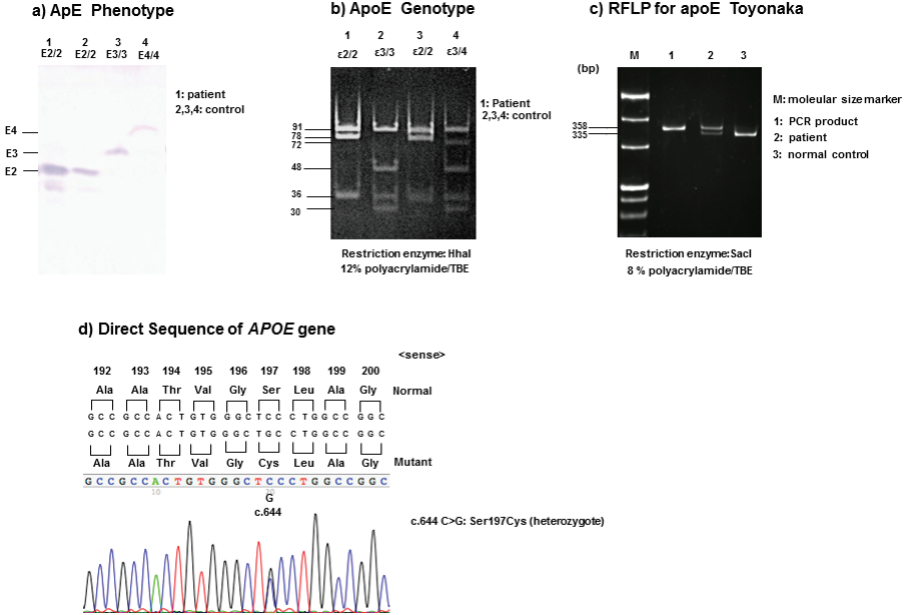
Phenotype, genotype, and DNA sequence of ApoE in this patient. a: ApoE phenotype analysis. The patient was identified as E2/2 (lane 1). Controls (lanes 2, 3, and 4) show E2/3, E3/3, and E3/4, respectively. b: APOE genotype analysis by RFLP using HhaI as a restriction enzyme. The patient was identified as ε2/2. Controls (lanes 2, 3, and 4) show ε2/2, ε3/4, and ε3/3, respectively. c: RFLP for ApoE Toyonaka using SacI as a restriction enzyme. Lanes M, 1, 2, and 3 show marker, PCR only, the patient (ApoE Toyonaka), and normal control, respectively. d: Sequence analysis of *APOE* gene. A heterozygous missense mutation (c.644 C>G) in exon 4 leads to an amino acid substitution Cys (TGC, lower) for Ser (TCC, upper) at codon 197.

Plasma ApoE phenotypes were analyzed by isoelectric focusing in polyacrylamide gel electrophoresis and immunoblotting analysis as previously reported [2, 3]. The patient’s sample showed the position of ApoE2/2 (Figure 4a, Lane 1). 

The *APOE* genotype was determined by restriction fragment length polymorphism (RFLP) analysis as described previously [2, 3]. The PCR products were digested with the restriction enzyme HhaI. Genotype ε2/2 in the patient was identified by 91- and 83-bp fragments (Figure 4b, Lane 1). The ApoE Toyonaka (Ser197Cys) as shown in Figure 4d was also confirmed by RFLP analysis digested with SacI (Figure 4c). Genomic DNA was amplified by polymerase chain reaction (PCR) using oligonucleotide primers, sense 5′-CGTGCGGGCCGCCACTGTGAGCT-3′ and antisense 5′-TCGCATGGCTGCAGGCTTCGGCGTTC-3′. The 335-bp fragment after cleavage by SacI suggested normal c.644C of ApoE in codon 197 (Lane 3). The 358- and 335-bp fragments after cleavage by SacI showed a heterozygous novel mutation of ApoE Toyonaka (Lane 2). Direct sequencing of *APOE* DNA was performed as described previously [7]. Based on the results, we identified a homozygous polymorphism at codon 158 (e2/2: c.526 C>T: Arg158Cys) (data not shown) and a heterozygous missense mutation (C to G) in exon 4 leading to an amino acid substitution Cys (TGC) for Ser (TCC) at codon 197 (c.644 C>G: p.S215C: Ser197Cys) (Figure 4d). All these findings were the same as that of a previous case of ApoE2/2 and ApoE Toyonaka showing MN-like glomerular histology [6]. 

### Clinical course after biopsy 

For hypertriglyceridemia, fibrate 400 mg/day was started. Obtaining the results of the IF and EM studies, which did not deny immune-related MN like lesions, PSL 10 mg/day and cyclosporine (CyA) 75 mg/day for NS were started. These treatments brought about a substantial improvement of hypertriglyceridemia and complete remission of NS by 300 days after admission. During maintenance therapy with oral PSL 5 mg and CyA 75 mg/day, he had cerebral infarction and aspiration pneumonia. After withdrawal of CyA, his NS relapsed with an increase of proteinuria more than 1 g/day. 

## Discussion 

In this report, a patient clinically presented with NS, and renal biopsy revealed segmental sclerosis and infiltration of foam cells in the capillary lumina in LM, similar to those of ApoE2 homozygote glomerulopathy with type III HLP [4]. In addition, spike formation was partially seen with PAM staining, and EM confirmed various EDDs in the subendothelial and subepithelial areas with apparent spike formation in a part of the GBM. These unique EDDs were proved to be formed by microbubbles in a highly-magnified EM. 

Type III HLP occurs in ~ 10% of the population who are homozygous for ApoE2. The ApoE2 allele is found in ~ 8% of the population, and ApoE2/2 occurs with a frequency of ~ 0.3% and 1% in Japanese [8] and Caucasian [9] populations, respectively. That only a minority of ApoE2 homozygotes develop type III HLP implies a multifactorial disorder. ApoE2 homozygote cases develop type III HLP when some genetic, hormonal, and environmental factors are added to ApoE2 mutation [10, 11]. Patients who present renal lipidosis with homozygous E2/2, also called ApoE2 homozygote glomerulopathy, are much rarer [4]. 

Although ApoE2 homozygote glomerulopathy is characterized by foam cell infiltration in the intracapillary and mesangial areas in general [4, 12, 13], Sakatsume et al. [14] reported a case showing subendothelial EDDs with slight subepithelial EDDs. Similar findings were presented in a few cases of LPG, the other ApoE-related glomerulopathy [15, 16]. In contrast to these cases, obvious subepithelial deposits forming spike formations of MN were recognized in the current case. However, these EDDs were different from those of immunogenetic MN because microbubbles were observed by high-power EM instead of immune deposits defined by IF. Intracapillary lipid deposits with positive Sudan III staining were also focally observed in our case, but these never showed the lamellar formation typically observed in LPG by EM. 

Recently, Fukunaga et al. [6] reported a case with homozygous E2/2 and ApoE Toyonaka that showed MN-like subepithelial EDDs. However, their case seemed different from MN, one of the representative immune diseases, because immunoglobulins and complements were not included in EDDs. Meanwhile, microbubbles were recognized by high-power EM. Although lipoprotein thrombi and foam cells were not observed in their case [6], characteristics of EDD were very similar to those of our case, and the interaction between a novel ApoE Toyonaka and homozygous E2/2 may contribute to the development of EDD similar to our case. 

The precise mechanism of how ApoE Toyonaka is involved in renal impairment is unknown. However, unlike other ApoE mutations associated with renal involvement, such as LPG and ApoE2 homozygote glomerulopathy, ApoE Toyonaka (Ser197Cys) is expressed in the hinge region (amino acid residues 192-215) [17], which stabilizes the connection between the N-terminal domain having LDL-receptor-binding region and the C-terminal domain having lipid-binding activity. Since the connection of the two domains in the hinge region is crucial for maintaining the lipoprotein-binding and tertiary structure of ApoE [18, 19], ApoE Toyonaka may modulate the functional capacity and structural disposition, and induce characteristic EDDs, which involve the accumulation of subepithelial deposits forming microbubbles. Although the current case had many foam cells characteristic of ApoE2 homozygote glomerulopathy unlike the case by Fukunaga et al. [6], this finding may be due to the different influences of ApoE Toyonaka in each case. However, the mutation in the hinge region has been unknown so far, and the hinge abnormality has not been studied yet. 

Until now, MN has been thought to occur only by the deposition of immune complex, but ApoE is a low molecular protein and may be involved in MN-like lesions due to the above-mentioned mechanism. A limitation of this case presentation is that we did not try the detection of ApoE as the component of EDD, but such studies have been done in similar cases by immunohistochemical technique [14] and tandem mass spectrometry [6]. Further case studies are needed to elucidate a more detailed mechanism. 

As the histology was similar to MN, our patient was treated with steroids and CyA as well as fibrates. As discussed above, it is unlikely that an immune mechanism was involved in this case, but it was of interest that complete remission was achieved by the administration of CyA. Apart from immunosuppressive effect, CyA is known to act directly on podocytes and contribute to the prompt reduction of urine protein [20]. In our case, such a mechanism may have played a role in the improvement of NS. 

On the other hand, as shown in our case, the effect of fibrates is more important for the glomerulopathy associated with type III HLP [4, 7, 14]. Moreover, LDL apheresis or plasma exchange may be considered in addition to fibrates if necessary. 

As another limitation, we did not perform the PLA2R test for the renal biopsy tissue or the serum, which would have been very helpful to substantiate the nonimmunologic nature of the EDDs. 

In conclusion, we encountered a patient with ApoE2 homozygote glomerulopathy showing EDD in both sides of the GBM. The novel mutation, ApoE Toyonaka with ApoE2/2 may contribute to subepithelial deposition forming MN-like lesions. Further accumulation and investigation of cases with the same combination of mutations are required. 

## Acknowledgment 

The authors express their sincere thanks for the supportive pathological analysis of Dr. Jun Kawai, Director of the Department of Pathology, Kansai Electric Power Hospital, Osaka and for the fruitful discussion and suggestion of Dr. Tatsuo Tsukamoto, Director, and the members of the Department of Nephrology and Dialysis, Kitano Hospital, Tazuke Kofukai Medical Research Institute, Osaka, Japan. 

## Funding 

None. 

## Conflict of interest 

The authors have no conflicts of interest to disclose.
